# Editorial

**DOI:** 10.1093/jhps/hnaa053

**Published:** 2020-10-09

**Authors:** Richard (Ricky) Villar

**Affiliations:** Editor-in-Chief, Journal of Hip Preservation Surgery

When ISHA first approached the *Journal of Hip Preservation Surgery*, and hence Oxford University Press, to ask about preparing a Supplement of the 2019 ISHA Annual Scientific Meeting in Madrid, it was easy to say ‘Yes’. After all, ISHA members, and certainly meeting attendees, form a major part of the readership of this journal. However, I had not expected the end-result to be so professional, so clear and, to put it frankly, so incredibly useful. Thanks to some really hard work by multiple authors, and some serious helmsmanship by Damian Griffin, aided by detailed input from so many who are involved with ISHA, this Supplement has turned out to be exceptional. When I read it for the first time, I realized I was looking at something different. I predicted that unlike so many other reports of annual meetings, this one will act as a major source of reference. The Supplement covers a huge spectrum of hip preservation surgery and carries just short of 120 references. For any of us writing a paper, the chances are we will find plenty of useful information here. It is also up to date, and a report of the views, findings and thoughts of the real frontrunners in modern-day hip preservation surgery. Well done to all those involved in bringing this Supplement together. Yet I suppose a top-rate product is to be expected. After all, the Madrid meeting was entitled, ‘Teamwork in hip preservation’.

Now, fellow readers, please remember to look at this, learn from it, and cite its contents wherever and whenever you can. You are looking at gold dust.

My very best wishes to you all.

